# Supplementary material for: Analysis of PPARγ Signaling Activity in Psoriasis
Source: Int J Mol Sci. 2021 Aug 10;22(16):8603. doi: 10.3390/ijms22168603 (PMC8395241; doi:10.3390/ijms22168603)
Supplement: Supplementary file 1 [file ijms-22-08603-s001.zip › Supplemental materials_Analysis of PPARg signaling activity in psoriasis/Pathway models/Models images and html files/Differentiation of psoriatic T cells/6372.html]

IL12B


# Protein IL12B

|  |  |
| --- | --- |
| URN | urn:agi-llid:3593 |
| Total Entities | 0 |
| Connectivity | 2324 |
| Name | IL12B |
| Description | interleukin 12B |
| Notes | This gene encodes a subunit of interleukin 12, a cytokine that acts on T and natural killer cells, and has a broad array of biological activities. Interleukin 12 is a disulfide-linked heterodimer composed of the 40 kD cytokine receptor like subunit encoded by this gene, and a 35 kD subunit encoded by IL12A. This cytokine is expressed by activated macrophages that serve as an essential inducer of Th1 cells development. This cytokine has been found to be important for sustaining a sufficient number of memory/effector Th1 cells to mediate long-term protection to an intracellular pathogen. Overexpression of this gene was observed in the central nervous system of patients with multiple sclerosis (MS), suggesting a role of this cytokine in the pathogenesis of the disease. The promoter polymorphism of this gene has been reported to be associated with the severity of atopic and non-atopic asthma in children. [provided by RefSeq, Jul 2008] |
| Primary Cell Localization | Extracellular |
| Class | Ligand |

---

|  |  |
| --- | --- |
| Pathway | Toll-like Receptors Act through MYD88-TIRAP Signaling |
|  | Dectin-1 (CLEC7A) Signaling |
|  | TLR4 Signaling in Leukocytes |
|  | CD8+ T-Cell Activation |
|  | Th1-Cell Differentiation |
|  | Toll-like Receptors in Sterile Inflammation |
|  | Macrophage M1 Lineage |
|  | CD16/CD14 Proinflammatory Monocyte Activation |
|  | AHR Signaling in M1 Macrophages Function |
|  | AXL Receptor Inhibits Macrophages and Dendritic Cells Function |
|  | Dendritic Cells Function in Atherosclerosis |
|  | Th1-Cells Activation and Proliferation in Atherosclerosis |
|  | Lymphocyte Mediated Myocardial Injury in Myocarditis |
|  | Inflammatory Reaction in Acne Vulgaris |
|  | Atopic Dermatitis |
|  | Proteins Involved in Psoriasis |
|  | Genes with Mutations Associated with Psoriasis |
|  | T-Cells Differentiation Block in Psoriasis |
|  | Dendritic Cells Function in Psoriasis |
|  | Th1-Cell Function in Systemic Lupus Erythematosus |
|  | Neutrophil and Macrophage Function in Systemic Lupus Erythematosus |
|  | T-Cell Cytotoxic Response against Melanocytes in Vitiligo |
|  | Toll-like Receptors in beta-Cell |
|  | Macrophage Function in Diabetes Mellitus Type 1 |
|  | TLR7 and TLR9 in Plasmacytoid Dendritic Cell |
|  | Proteins Involved in Diabetes Mellitus Type 1 |
|  | Conventional Dendritic Cell Role in Diabetes Mellitus Type 1 |
|  | Proteins Involved in Hashimoto's Thyroiditis |
|  | CD4+T-Cell Response in Celiac Disease |
|  | Th1-Cell Activation in Crohn's Disease |
|  | Th17-Cell Activation in Crohn's Disease |
|  | Proteins Involved in Inflammatory Bowel Diseases |
|  | Dendritic Cell Dysfunction in Crohn's disease |
|  | DupA Induced IL12 Production in Helicobacter Pylori Infection |
|  | Dendritic Cell Function in Ulcerative Colitis |
|  | Follicular Lymphoma |
|  | Il17 Signaling Related Neutrophilia in Asthma |
|  | Th17-Cell and Th1 Immune Responsein Psoriatic Arthritis |
|  | Proteins Involved in Psoriatic Arthritis |
|  | Genes with Mutations Associated with Psoriatic Arthritis |
|  | Proteins with Altered Expression in Psoriatic Arthritis |
|  | Proteins Involved in Glioblastoma |
|  | IL6/IL12 Signaling Activates Immune System in Multiple Sclerosis |
|  | CD8 -> STAT Expression Targets |
|  | CD80 -> STAT Expression Targets |
|  | CSF2 -> STAT Expression Targets |
|  | ADCYAP1 Expression Targets |
|  | IL12R -> STAT Signaling |
|  | IL23R -> STAT3/NF-kB Signaling |
|  | IL12R -> NF-kB/NFATC Signaling |
|  | IL12B Expression Targets |
|  | Elevated Receptors -> Expression Targets in Lymphoid System and Blood |
|  | Immunoglobulin Superfamily -> Expression Targets in Lymphoid System and Blood |
|  | CD40 -> Expression Targets in Thymus |
|  | CD40 -> Expression Targets in Lymph Node |
|  | Elevated Receptors -> Expression Targets in Thymus |
|  | PRL/PRLR Expression Targets |
|  | PRL/GHR -> STAT Expression Targets |
|  | PRL/GHR -> NF/kB/ELK/SRF/MYC Expression Targets |
|  | TCR -> CREB/CREBBP/ATF Expression Targets |
|  | TCR -> NF-kB Expression Targets |
|  | TCR -> NFAT Expression Targets |
|  | TCR -> STAT Expression Targets |
|  | TCR -> AP-1 Expression Targets |
|  | TGFB1-TGFBR1/AP-1 Expression Targets |
|  | TNF -> NF-kB Expression Targets |
|  | CD40LG -> STAT Expression Targets |
|  | CD40LG -> NF-kB/ELK/SRF -> CREB/NFATC Expression Targets |
|  | TLR3 Expression Targets |
|  | TLR4 -> AP-1 Expression Targets |
|  | TLR7 Expression Targets |
|  | TLR1 -> 2/6 Expression Targets |
|  | IL1B Expression Targets |
|  | TLR4 -> NF-kB/IRF Expression Targets |
|  | TLR9 Expression Targets |
|  | ROS in Triggering Vascular Inflammation |
|  | PPAR Psoriasis |
|  | pprarg neg, uknown targets, ps-positive |
|  | prarg neg,ukn expres targets, ps-positive |
|  | PPARG negative targets |
|  | pparg expr target\_regulators |
|  | Neighbors of disease exacerbation |
|  | Neighbors of mortality |
|  | Neighbors of survival rate |
|  | Neighbors of bacterial load |
|  | Neighbors of graft survival |
|  | Neighbors of therapeutic efficacy |
|  | Neighbors of lethality |
|  | Neighbors of recurrence-free survival |
|  | Neighbors of tumor response |
|  | Neighbors of long-term survival |
|  | Neighbors of treatment outcome |
|  | Neighbors of body weight |
|  | Neighbors of morbidity |
|  | Neighbors of cancer risk |
|  | Neighbors of cancer incidence |
|  | Neighbors of T-cell count |
|  | Neighbors of skin thickness |
|  | Neighbors of disease exacerbation |
|  | Neighbors of mortality |
|  | Neighbors of survival rate |
|  | Neighbors of bacterial load |
|  | Neighbors of graft survival |
|  | Neighbors of therapeutic efficacy |
|  | Neighbors of lethality |
|  | Neighbors of recurrence-free survival |
|  | Neighbors of tumor response |
|  | Neighbors of long-term survival |
|  | Neighbors of treatment outcome |
|  | Neighbors of body weight |
|  | Neighbors of morbidity |
|  | Neighbors of cancer risk |
|  | Neighbors of cancer incidence |
|  | Neighbors of T-cell count |
|  | Neighbors of skin thickness |
|  | Neighbors of cell development |
|  | Neighbors of immune response |
|  | Neighbors of T-cell development |
|  | Neighbors of inflammatory response |
|  | Neighbors of angiogenesis |
|  | Neighbors of innate immune response |
|  | Neighbors of cell function |
|  | Neighbors of macrophage activation |
|  | Neighbors of cell formation |
|  | Neighbors of adaptive immune response |
|  | Neighbors of immunity |
|  | Neighbors of chemotaxis |
|  | Neighbors of dendritic cell differentiation |
|  | Neighbors of cell phenotype |
|  | Neighbors of T-cell proliferation |
|  | Neighbors of cell adhesion |
|  | Neighbors of T-cell activation |
|  | Neighbors of tumor growth |
|  | Neighbors of dendritic cell development |
|  | Neighbors of cell count |
|  | Neighbors of pregnancy |
|  | Neighbors of neutrophil recruitment |
|  | Neighbors of cell migration |
|  | Neighbors of hepatic regeneration |
|  | Neighbors of phagocytosis |
|  | Neighbors of immune system activation |
|  | Neighbors of hemopoiesis |
|  | Neighbors of ossification |
|  | Neighbors of T-cell function |
|  | Neighbors of immune system function |
|  | Neighbors of lung development |
|  | Neighbors of autophagy |
|  | Neighbors of T-cell response |
|  | Neighbors of tumor immunity |
|  | Neighbors of neuroprotection |
|  | Neighbors of cell growth |
|  | Neighbors of sensitization |
|  | Neighbors of hemato-encephalic barrier |
|  | Neighbors of cellular immune response |
|  | Neighbors of cell differentiation |
|  | Neighbors of psoriasis |
|  | Neighbors of inflammatory disease |
|  | Neighbors of rheumatoid arthritis |
|  | Neighbors of fibrosis |
|  | Neighbors of colitis |
|  | Neighbors of arthritis |
|  | Neighbors of inflammatory bowel disease |
|  | Neighbors of metastasis |
|  | Neighbors of carcinogenesis |
|  | Neighbors of multiple sclerosis |
|  | Neighbors of chronic inflammation |
|  | Neighbors of inflammation |
|  | Neighbors of diabetes mellitus |
|  | Neighbors of asthma |
|  | Neighbors of pneumonia |
|  | Neighbors of leukocyte infiltration |
|  | Neighbors of airway inflammation |
|  | Neighbors of hepatitis |
|  | Neighbors of insulin resistance |
|  | Neighbors of infection |
|  | Neighbors of sepsis |
|  | Neighbors of death |
|  | Neighbors of type 1 diabetes |
|  | Neighbors of encephalomyelitis |
|  | Neighbors of autoimmune disease |
|  | Neighbors of dermatitis |
|  | Neighbors of heart disease |
|  | Neighbors of lung injury |
|  | Neighbors of immunopathology |
|  | Neighbors of hepatocellular carcinoma |
|  | Neighbors of autoimmunity |
|  | Neighbors of acute lung injury |
|  | Neighbors of experimental autoimmune encephalomyelitis |
|  | Neighbors of systemic lupus erythematosus |
|  | Neighbors of melanoma |
|  | Neighbors of collagen-induced arthritis |
|  | Neighbors of pulmonary fibrosis |
|  | Neighbors of cardiovascular disease |
|  | Neighbors of cancer |
|  | Neighbors of type 2 diabetes |
|  | Neighbors of neoplasm |
|  | Neighbors of respiratory hypersensitivity |
|  | Neighbors of liver disease |
|  | Neighbors of allograft rejection |
|  | Neighbors of epithelial cell |
|  | Neighbors of keratinocyte |
|  | Neighbors of monocyte |
|  | Neighbors of alveolar macrophage |
|  | Neighbors of macrophage |
|  | Neighbors of adipocyte |
|  | Neighbors of activated monocyte |
|  | Neighbors of fibroblast |
|  | Neighbors of airway epithelial cell |
|  | Neighbors of PBMC |
|  | Neighbors of microglia |
|  | Neighbors of mononuclear cell |
|  | Neighbors of endothelial cell |
|  | Neighbors of dendritic cell |
|  | Neighbors of Kuppfer cell |
|  | Neighbors of inflammatory cell |
|  | Neighbors of bone marrow macrophage |
|  | Neighbors of peritoneal macrophage |
|  | Neighbors of neutrophil |
|  | Neighbors of astrocyte |
|  | Neighbors of bone marrow derived dendritic cell |
|  | Neighbors of mononuclear phagocyte |
|  | Neighbors of immunocompetent cell |
|  | Neighbors of granulocyte |
|  | Neighbors of myeloid cell |
|  | Neighbors of stem cell |
|  | Neighbors of sinusoidal cell |
|  | Neighbors of B-cell |
|  | Neighbors of leukocyte |
|  | Neighbors of insulin-secreting cell |
|  | Neighbors of T-cell |
|  | Neighbors of M1 macrophage |
|  | Neighbors of spleen cell |
|  | Neighbors of conventional dendritic cell |
|  | Neighbors of antigen-presenting cell |
|  | Neighbors of phagocyte |
|  | Neighbors of lymphocyte |
|  | Neighbors of Langerhans cell |
|  | Neighbors of M2 macrophage |
|  | Neighbors of peritoneum cell |
|  | Neighbors of blood cell |
|  | Neighbors of skin cell |
|  | Neighbors of lymph node cell |
|  | Neighbors of lung |
|  | Neighbors of kidney |
|  | Neighbors of skin |
|  | Neighbors of intestine |
|  | Neighbors of lymph node |
|  | Neighbors of colon |
|  | Neighbors of gastrointestinal tract |
|  | Neighbors of bone |
|  | Neighbors of brain |
|  | Neighbors of islets of Langerhans |
|  | Neighbors of pancreas |
|  | Neighbors of spleen |
|  | Neighbors of alveolus |
|  | Neighbors of cornea |
|  | Neighbors of lymph node of mesentery |
|  | Neighbors of thymus gland |
|  | Neighbors of draining lymph node |
|  | Neighbors of NF-kB family |
|  | Neighbors of oxidized LDL |
|  | Neighbors of JNK |
|  | Neighbors of Jun/Fos |
|  | Neighbors of NFKBI |
|  | Neighbors of PKC |
|  | Neighbors of PI3K |
|  | Neighbors of STAT family |
|  | Neighbors of mitogen-activated protein kinase |
|  | Neighbors of IFNAR ligand |
|  | Neighbors of TLR |
|  | Neighbors of cytokine |
|  | Neighbors of interferon |
|  | Neighbors of JAK |
|  | Neighbors of MEK1/2 |
|  | Neighbors of ERK1/2 |
|  | Neighbors of growth factor |
|  | Neighbors of PKA |
|  | Neighbors of ovalbumin |
|  | Neighbors of NF-AT family |
|  | Neighbors of beta adrenoceptor |
|  | Neighbors of chorionic gonadotropin |
|  | Neighbors of IKBK |
|  | Neighbors of NAD(P)H oxidase |
|  | Neighbors of IgG |
|  | Neighbors of AMPK |
|  | Neighbors of LXR |
|  | Neighbors of HDL |
|  | Neighbors of p38 |
|  | Neighbors of histone deacetylase |
|  | Neighbors of IL1R |
|  | Neighbors of HIF-1 |
|  | Neighbors of IL12 |
|  | Neighbors of Notch |
|  | Neighbors of prostaglandin-endoperoxide synthase |
|  | Neighbors of lectin |
|  | Neighbors of fibrinogen |
|  | Neighbors of NOS |
|  | Neighbors of proteasome endopeptidase complex |
|  | Neighbors of histone H3 |
|  | Neighbors of alpha2 adrenoceptor |
|  | Neighbors of PP2A |
|  | Neighbors of SAPK |
|  | Neighbors of phospholipase |
|  | Neighbors of CD3 complex |
|  | Neighbors of TLR1/2 heterodimer |
|  | Neighbors of TORC1 |
|  | Neighbors of histone |
|  | Neighbors of neuropeptides |
|  | Neighbors of histone acetyltransferase |
|  | Neighbors of caspase |
|  | comon ps\_pos, pprarg\_neg targets |
|  | 1\_Inflammation in keratinocytes in the pathology of acne |
|  | 1\_Differentiation of psoriatic T cells |
|  | 1\_Defects in response to pathogens in the gut promotes inflammation in Crohn's disease |
|  | 2\_Intestine epithelial cells drive immunological responses in ulcerative colitis |
|  | 4\_1\_Polymorphisms associated with inflammatory bowel diseases |
|  | 1\_Antigen-presenting cells promote islet dysfunction and immune system activation in T1D |
|  | Figure 4\_Diabetes mellitus Type 1: conventional dendritic cell |
|  | Differentiation of psoriatic T cells |

---

|  |  |
| --- | --- |
| Group | Genes with Mutations Associated with Psoriasis |
|  | Genes with Mutations Associated with Inflammatory Bowel Diseases |
|  | Genes with Mutations Associated with Psoriatic Arthritis |

---

|  |  |
| --- | --- |
| MedScan ID | 3593 |

---

|  |  |
| --- | --- |
| LocusLink ID | 3593 |
|  | 16160 |
|  | 64546 |

---

|  |  |
| --- | --- |
| Alias | Interleukin-12 beta |
|  | BHR I |
|  | cytotoxic lymphocyte maturating factor 2 |
|  | interleukin IL23, subuint p40 |
|  | IL23, subuint p40 |
|  | interleukin 12B (natural killer cell stimulatory factor 2, cytotoxic lymphocyte maturation factor 2, p40) |
|  | BHR1 |
|  | IL-12/23 p40 |
|  | interleukin-12 p40 subunit |
|  | interleukin 12 beta subunit |
|  | IL-12 p40 |
|  | I12B\_MOUSE |
|  | IL12B |
|  | interleukin IL-12 p40 |
|  | Bronchial hyperresponsiveness-1 (bronchial asthma) |
|  | natural killer cell stimulatory factor-2 |
|  | RP23-388G23.1 |
|  | interleukin 12B |
|  | bronchial hyperresponsiveness 1 |
|  | Cytotoxic lymphocyte maturation factor 40 kDa subunit |
|  | cytotoxic lymphocyte maturating factor II |
|  | cytotoxic lymphocyte maturating factor 2, p40 |
|  | natural killer cell stimulatory factor, 40 kD subunit |
|  | bronchial hyperresponsiveness-I |
|  | cytotoxic lymphocyte maturation factor II |
|  | interleukin 12, p40 |
|  | OTTMUSP00000005619 |
|  | bronchial hyperresponsiveness I |
|  | I12B |
|  | Cytotoxic lymphocyte maturating factor 40 kDa subunit |
|  | interleukin IL12, subunit p40 |
|  | cytotoxic lymphocyte maturation factor 2, p40 |
|  | CLMF P40 |
|  | interleukin IL-12-specific subunit p40 |
|  | NK cell stimulatory factor chain II |
|  | CLMF2 |
|  | cytotoxic lymphocyte maturation factor 2 |
|  | Interleukin IL12 beta |
|  | natural killer cell stimulatory factor II |
|  | I12B\_HUMAN |
|  | interleukin IL12b |
|  | IL-23 p40 |
|  | bronchial hyperresponsiveness-1 |
|  | NKSF2 |
|  | OTTHUMP00000160820 |
|  | IL12, subunit p40 |
|  | NK cell stimulatory factor chain 2 |
|  | IL-23 subunit p40 |
|  | interleukin 12 subunit p40 |
|  | interleukin-12 subunit beta |
|  | IL-12-specific subunit p40 |
|  | IL-12 p40 subunit |
|  | CLMF |
|  | NKSF |
|  | IMD28 |
|  | IMD29 |
|  | IL-12B |
|  | IL-12 subunit p40 |
|  | interleukin-12 beta chain |
|  | p40 |
|  | Il12p40 |
|  | Il-12p40 |
|  | Il12 |
|  | interleukin-12 p40 |

---

|  |  |
| --- | --- |
| GO ID | 0005125 |
|  | 0019955 |
|  | 0004896 |
|  | 0008083 |
|  | 0042164 |
|  | 0005143 |
|  | 0045519 |
|  | 0046982 |
|  | 0042803 |
|  | 0035744 |
|  | 0042088 |
|  | 0042093 |
|  | 0007050 |
|  | 0016477 |
|  | 0008283 |
|  | 0071346 |
|  | 0071222 |
|  | 0019221 |
|  | 0050829 |
|  | 0042832 |
|  | 0051607 |
|  | 0042095 |
|  | 0035722 |
|  | 0038155 |
|  | 0030101 |
|  | 0002323 |
|  | 0044130 |
|  | 0002862 |
|  | 0032693 |
|  | 0032700 |
|  | 0048662 |
|  | 1901224 |
|  | 0051135 |
|  | 0051142 |
|  | 0001916 |
|  | 0042102 |
|  | 0002827 |
|  | 2000330 |
|  | 2000318 |
|  | 0042104 |
|  | 0010536 |
|  | 0045785 |
|  | 0002230 |
|  | 0032725 |
|  | 0050729 |
|  | 0045078 |
|  | 0032729 |
|  | 0032733 |
|  | 0032735 |
|  | 0032740 |
|  | 0050671 |
|  | 0043382 |
|  | 0032946 |
|  | 0032816 |
|  | 0002860 |
|  | 0032819 |
|  | 0045672 |
|  | 0034393 |
|  | 0034105 |
|  | 0032760 |
|  | 0042531 |
|  | 0042035 |
|  | 0042509 |
|  | 0010224 |
|  | 0019233 |
|  | 0019953 |
|  | 0005829 |
|  | 0005788 |
|  | 0009897 |
|  | 0005576 |
|  | 0005615 |
|  | 0043514 |
|  | 0070743 |
|  | 0031906 |
|  | 0043235 |
|  | 0005126 |
|  | 0007166 |
|  | 0010033 |
|  | 0009986 |
|  | 0005737 |
|  | 0016020 |
|  | 0032609 |
|  | 0010469 |
|  | 0042346 |
|  | 0010535 |
|  | 0042517 |
|  | 0042520 |
|  | 0042523 |
|  | 0042510 |
|  | 0004871 |
|  | 0007165 |

---

|  |  |
| --- | --- |
| KEGG ID | hsa:3593 |
|  | mmu:16160 |
|  | rno:64546 |

---

|  |  |
| --- | --- |
| Organism | Homo sapiens {Organism urn:agi-taxid:9606} |
|  | Mus musculus {Organism urn:agi-taxid:10090} |
|  | Rattus norvegicus {Organism urn:agi-taxid:10116} |
|  | Mus musculus |
|  | Homo sapiens |
|  | Rattus norvegicus |

---

|  |  |
| --- | --- |
| Mouse chromosome position | 11 25.94 cM |
|  | 11 19.0 cM |

---

|  |  |
| --- | --- |
| OMIM ID | 161561 |
|  | 614890 |
|  | 600807 |
|  | 209950 |
|  | 177900 |

---

|  |  |
| --- | --- |
| Rat chromosome position | 10q21 |

---

|  |  |
| --- | --- |
| Hugo ID | 5970 |
|  | HGNC:5970 |

---

|  |  |
| --- | --- |
| Human chromosome position | 5q33.3 |
|  | 5q31.1-q33.1 |

---

|  |  |
| --- | --- |
| Swiss-Prot Accession | P29460 |
|  | P29460.1 |
|  | P43432 |
|  | Q3ZAX5 |
|  | P43432.1 |
|  | Q9R278 |
|  | E9PU71 |
|  | Q9QUM1 |
|  | Q8N0X8 |
|  | Q62712 |

---

|  |  |
| --- | --- |
| PIR ID | A38957 |
|  | I72789 |

---

|  |  |
| --- | --- |
| GenBank ID | NC\_000005 |
|  | NM\_002187 |
|  | NP\_002178 |
|  | NG\_009618 |
|  | AC011418 |
|  | AF512686 |
|  | AAM34792 |
|  | AY008847 |
|  | AAG32620 |
|  | AY064126 |
|  | CH471062 |
|  | EAW61576 |
|  | JA104591 |
|  | CCA63965 |
|  | KP140671 |
|  | AJQ18452 |
|  | KP140688 |
|  | KP140689 |
|  | KP140690 |
|  | KP140691 |
|  | KP140692 |
|  | KP140693 |
|  | KP140694 |
|  | KP140695 |
|  | KP140699 |
|  | KP140700 |
|  | U89323 |
|  | AF180563 |
|  | AAD56386 |
|  | AY046592 |
|  | AAL05890 |
|  | AY046593 |
|  | AAL05891 |
|  | BC067498 |
|  | AAH67498 |
|  | BC067499 |
|  | AAH67499 |
|  | BC067500 |
|  | AAH67500 |
|  | BC067501 |
|  | AAH67501 |
|  | BC067502 |
|  | AAH67502 |
|  | BC074723 |
|  | AAH74723 |
|  | EF173865 |
|  | ABM53138 |
|  | M65272 |
|  | AAA35695 |
|  | M65290 |
|  | AAA59938 |
|  | P29460 |
|  | NC\_000077 |
|  | NM\_001303244 |
|  | NP\_001290173 |
|  | AH004859 |
|  | AAB37383 |
|  | AL607030 |
|  | AL669944 |
|  | CH466575 |
|  | EDL33847 |
|  | EDL33848 |
|  | D63333 |
|  | AF128214 |
|  | AAF22555 |
|  | AF128215 |
|  | AAF22556 |
|  | AK162981 |
|  | BAE37146 |
|  | BC103608 |
|  | AAI03609 |
|  | BC103609 |
|  | AAI03610 |
|  | BC103610 |
|  | AAI03611 |
|  | BC103614 |
|  | AAI03615 |
|  | BY154173 |
|  | M86671 |
|  | AAA39296 |
|  | P43432 |
|  | NC\_005109 |
|  | XM\_006246147 |
|  | XP\_006246209 |
|  | NM\_022611 |
|  | NP\_072133 |
|  | AC\_000078 |
|  | AABR07029474 |
|  | AAHX01063521 |
|  | CH473948 |
|  | EDM04146 |
|  | EDM04147 |
|  | AF133197 |
|  | AAD24190 |
|  | U16674 |
|  | AAA52229 |
|  | NC\_018916 |
|  | AMYH02012586 |
|  | HQ447568 |
|  | ADQ32054 |
|  | AC\_000033 |
|  | AAHY01092921 |
|  | AC\_000137 |
|  | ABBA01044239 |
|  | XM\_006532306 |
|  | XP\_006532369 |
|  | NM\_008352 |
|  | NP\_032378 |
|  | S82421 |
|  | S82422 |
|  | S82424 |
|  | S82425 |
|  | S82426 |
|  | CAM20288 |
|  | CAM22832 |
|  | NT\_023133 |
|  | NW\_001838954 |
|  | NW\_922784 |
|  | AC\_000048 |
|  | Q8N0X8 |
|  | NT\_096135 |
|  | NW\_001030461 |
|  | Q3ZAX5 |
|  | NW\_047334 |
|  | NW\_001084656 |
|  | Q62712 |
|  | Q9R278 |

---

|  |  |
| --- | --- |
| Swiss-Prot ID | IL12B\_HUMAN |
|  | IL12B\_MOUSE |
|  | Q9R278\_RAT |
|  | Q3ZAX5\_MOUSE |

---

|  |  |
| --- | --- |
| Cell Localization | Secreted |
|  | Extracellular |

---

|  |  |
| --- | --- |
| Ensembl ID | ENSG00000113302 |
|  | ENSP00000231228.2 |
|  | ENST00000231228.2 |
|  | ENSMUSG00000004296 |
|  | ENSMUSP00000099860.3 |
|  | ENSMUST00000102796.9 |
|  | ENSRNOG00000004380 |
|  | ENSRNOP00000005898.5 |
|  | ENSRNOT00000005898.6 |
|  | ENSP00000231228 |
|  | ENST00000231228 |
|  | ENSMUSP00000099860 |
|  | ENSMUST00000102796 |
|  | ENSRNOP00000005898 |
|  | ENSRNOT00000005898 |

---

|  |  |
| --- | --- |
| MGI ID | MGI:96540 |
|  | 96540 |

---

|  |  |
| --- | --- |
| RGD ID | 628704 |

---

|  |  |
| --- | --- |
| Unigene ID | Hs.674 |
|  | Mm.239707 |
|  | Rn.48686 |

---

|  |  |
| --- | --- |
| Homologene ID | 1648 |

---

|  |  |
| --- | --- |
| Shape | Rhomb |

---

|  |  |
| --- | --- |
| IPI ID | IPI00120356 |
|  | IPI00021798 |
|  | IPI00207823 |

---
